# Appendicitis after colonoscopy—a case report, literature review, and synopsis of the pitfalls in diagnosis

**DOI:** 10.1093/jscr/rjae362

**Published:** 2024-05-30

**Authors:** Austin Milton, Bradley Cox, Michael Charles, Zhamak Khorgami

**Affiliations:** Surgery Department, University of Oklahoma, School of Community Medicine, Tulsa, OK 74135, United States; Surgery Department, University of Oklahoma, School of Community Medicine, Tulsa, OK 74135, United States; Surgery Department, University of Oklahoma, School of Community Medicine, Tulsa, OK 74135, United States; Surgery Department, University of Oklahoma, School of Community Medicine, Tulsa, OK 74135, United States

**Keywords:** appendicitis, colonoscopy, complication, insufflation, barotrauma, fecalith, appendicolith

## Abstract

A case is described in which appendicitis presented in a 73-year-old woman the day after a colonoscopy. Possible mechanisms for appendicitis aggravated by colonoscopy include barotrauma, irritation by residual glutaraldehyde type solution used for cleaning the endoscope, fecalith, and/or appendicolith being pushed into the orifice of the appendix by insufflation during the colonoscopy. This rare complication is likely most often unavoidable due to the pressure required to properly visualize the colon (which typically ranges from 9 to 57 mmHg) and the manipulation required to visualize and cannulate the ileocecal valve. Physicians should consider possibility of acute appendicitis after colonoscopy when evaluating abdominal pain after a recent colonoscopy.

## Introduction

Appendicitis after colonoscopy is a rare complication. The literature regarding its incidence is inconsistent, and risk-factors are unclear [1–3]. We present a 73-year-old female requiring appendectomy the day after a diagnostic colonoscopy.

## Case

A 73-year-old female presented to the emergency department (ED) with a 1-d history of right lower quadrant (RLQ) pain, nausea, and reduced appetite starting the morning after a routine colonoscopy. Her history includes atrial fibrillation, cholecystectomy, hysterectomy, and metastatic endometrial cancer currently treated with radiation (on a hiatus at presentation) and maintenance chemotherapy of lenvatinib and pembrolizumab.

**Figure 1:**
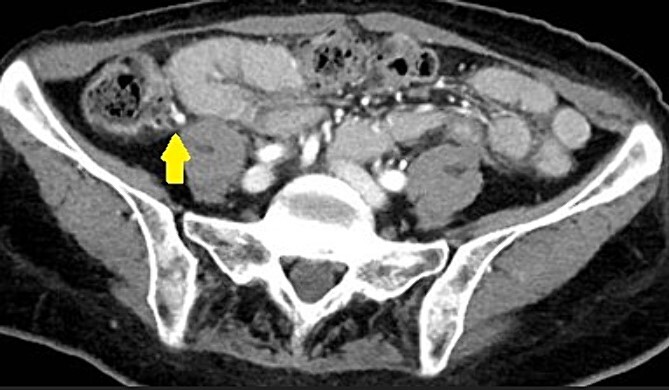
CT scan demonstrating an appendicolith (proven by pathology to be fecalith) at the base of the appendix.

Upon arrival, she was non-toxic in appearance. She was afebrile and her blood pressure was 195/83 mmHg and lowered to 150’s/80’s with pain control in the ED. Vital signs were otherwise stable. Abdomen was soft and non-distended and had a positive McBurney’s sign. There was exquisite RLQ tenderness to palpation. Other physical exams were normal. A urinalysis, complete blood count, and complete metabolic panel were unremarkable. White blood cell count was 5800 per microliter. A computed tomography (CT) scan (Fig. 1) was obtained and showed appendicitis with an appendicolith.

Piperacillin plus tazobactam for suspected appendicitis and ondansetron for nausea were initiated. She was taken for a laparoscopic appendectomy. The appendix was inflamed but not perforated, adhered to the lateral aspect of the ascending colon, and contained an appendicolith. The bowl and mesentery within the pelvis had chronic changes consistent with radiation enteritis. A gastrointestinal anastomosis stapler was used to divide the base of the appendix. A harmonic scalpel then freed the attachments. There were no intraoperative complications. The pathological specimen was confirmed appendicitis and periappendicitis with fecalith. It measured 4.6 cm long × 1.5 cm diameter with a lumen up to 0.5 cm.

**Figure 2:**
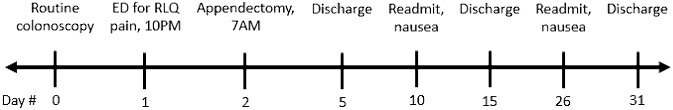
Timeline of the case from colonoscopy to final discharge.

She recovered well, tolerated regular diet and ambulated the evening after surgery. She had mild ileus after surgery. An x-ray on postoperative Day 2 revealed mildly distended bowel and no acute findings. She had a bowel movement on postoperative Day 2 and was discharged on postoperative Day 3. She had two readmissions for nausea, vomiting, and poor oral intake: 8 and 24 d after surgery. Each was 5-d duration. She remained hemodynamically stable with minimal abdominal tenderness. She was given antiemetics and diet was advanced as tolerated. She had experienced similar nausea without hospitalization while on chemotherapy prior to surgery. Figure 2 displays the case timeline.

## Discussion

Post-colonoscopy appendicitis presents the same as spontaneous appendicitis: RLQ pain localizing to McBurney’s point, fever, leukocytosis, nausea, and anorexia [4]. Appendicitis status post colonoscopy may not be considered, and this may delay appropriate imaging. Ultrasound has accuracy between 71 and 97% while CT scan has accuracy between 93 and 98% for appendicitis [5]. CT scan may become the preferred imaging modality when patients have comorbidities such as scarring [6].

Appendicitis after colonoscopy was first reported in London in 1988 when a gastrografin enema performed to rule out perforation in a 35-year-old male 8 h after colonoscopy revealed appendicitis [7]. Perforation is a major but rare complication of colonoscopy, with occurrence cited in the literature ranging from 0.005% in screening colonoscopy, and up to 5% after therapeutic colonoscopy [2, 8–10]. The incidence of perforation is thought to be unchanged in the last 15 years despite a focus on colonoscopy training [9, 11]. This may be because the high risk conditions have become more common among colonoscopy patients [9]. Other rare complications of colonoscopy that may be considered on a differential with appendicitis include post-polypectomy syndrome, splenic injury, small bowel perforation, cecal volvulus, and mesenteric ischemia [6, 12]. Significant pain after a colonoscopy should warrant strong consideration of a CT scan because it is sensitive for many of these findings [12].

A study has shown an odds ratio of 4.5 for appendicitis leading to appendectomy within the first week after a colonoscopy compared with appendectomies in the weeks that follow [1]. This study showed a rate of 1 in 58 000 cases presenting with appendicitis requiring appendectomy after a colonoscopy, excluding cases where appendicitis symptoms were present before the colonoscopy [1]. Without excluding those cases, the rate is 1 in 33 000. Other reports cite the rate of appendicitis after colonoscopy as 1 in 10 000 or higher [2, 3]. Appendicitis after colonoscopy is likely underdiagnosed because it may resolve with empiric antibiotics.

The mechanism of appendicitis after colonoscopy is unclear. Several mechanisms have been proposed in the literature including barotrauma, irritation by residual glutaraldehyde type solution used for cleaning the endoscope, fecalith, and/or appendicolith being pushed into the orifice of the appendix by insufflation during the colonoscopy [2]. Preexisting appendiceal inflammation, mucosal irritation induced by the colonoscope, and even direct intubation of the appendix by the scope may contribute to cases of appendicitis after colonoscopy [4].

Inverted appendix, also called appendiceal intussusception, is an appendix which protrudes into the lumen of the colon and can mimic a polyp during colonoscopy. There have been cases of ‘polypectomy’ of inverted appendices [13–15]. The pathophysiology of inverted appendices can shed light on possible causes of appendicitis after colonoscopy. Inverted appendix may be caused by fecalith, inflammation, endometriosis, mucocele, or appendiceal neoplasms [16]. These features could predispose to appendicitis; and colonoscopists should be cautious to watch for signs of appendicitis in patients who have these comorbidities.

This rare complication is likely unavoidable due to the pressure required to properly visualize the colon (which typically ranges from 9 to 57 mmHg) and the manipulation required to visualize and cannulate the cecum and ileocecal valve [17–19]. Physicians performing colonoscopies should consider mentioning appendicitis as a complication during the consent process for the procedure.
